# Master–apprentice technique for improved learning of endoscopic closure of defects using the reopenable clip-over-line method

**DOI:** 10.1055/a-2463-5784

**Published:** 2024-11-26

**Authors:** Akihiro Maruyama, Tatsuma Nomura, Hirotaka Takeshima, Hiroshi Nakayabu, Hiroki Kato, Shintaro Tominaga, Makoto Kobayashi

**Affiliations:** 1 37036 Gastroenterology, Yokkaichi Municipal Hospital, Yokkaichi, Japan; 2 36951 Gastroenterology, Suzuka General Hospital, Suzuka, Japan

Various methods for endoscopic closure of defects have been recently developed 1 . The reopenable clip-over-line method (ROLM) is a novel defect closure technique 2 3 4 , and we have developed a master–apprentice approach to improve training in this method 5 .

In the master–apprentice approach, a second operator (the “master” endoscopist) handles the device that is inserted into the accessory channel ( Fig. 1 , Video 1 ). This allows the “apprentice” endoscopist to focus on finding a point for clip placement on one edge of the defect while holding the endoscope and receiving instructions from the master endoscopist.

**Fig. 1:**
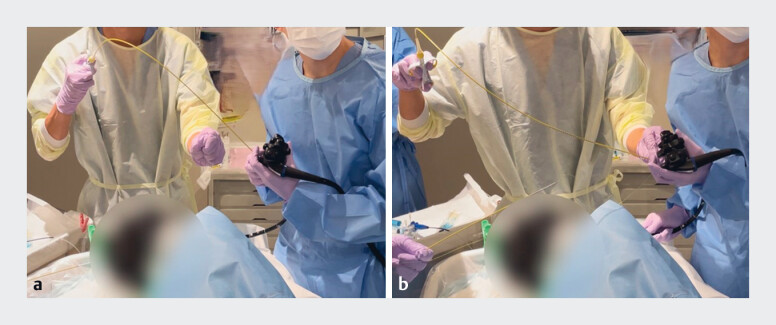
The master–apprentice approach for the reopenable clip-over-line method (ROLM). **a** In an ROLM procedure the clip is usually inserted while the endoscopist grasps the line with the little finger of the left hand. The endoscopist then handles the clip applicator using the right hand, which requires practice because the right hand is away from the scope. **b** In the master–apprentice method, the master endoscopist handles the clip device. while the apprentice endoscopist holds the endoscope and locates the appropriate clip placement site under guidance from the master. The master endoscopist then inserts and withdraws the clip applicator such that the apprentice can manipulate the scope precisely. The distance between the tip of the scope and area to be grasped can be correctly guided by the master endoscopist.

Reopenable clip-over-line method (ROLM) for endoscopic defect closure, using the master–apprentice technique to improve the learning curve.Video 1

Our patient developed a 40-mm mucosal defect after gastric endoscopic submucosal dissection (ESD). Complete defect closure was achieved using the ROLM and the master–apprentice approach ( Fig. 2 ). Initially, the master endoscopist handled the scope and indicated to the apprentice endoscopist where the reopenable clip should be placed. The scope was then given to the apprentice. The master then deployed the first clip, with attached line, at the defect edge on the anal-most side. The free end of the line had been passed through the hole in one jaw of a second clip, and the master endoscopist inserted this second reopenable clip over the line through the accessory channel. He firmly grasped the line to keep it sufficiently taut. When the clip appeared in the endoscopic view, the line was released, the clip device was pulled slightly, and then reinserted to maintain a distance from the first clip, allowing free manipulation of the second clip. The master endoscopist rotated the second reopenable clip (that had the line passed through the hole in the jaw), allowing the apprentice endoscopist to maneuver to an appropriate endoscopic view of the defect edges. As the master endoscopist performed most of the clip manipulation, the apprentice endoscopist could assist in closing the defect by simply obtaining the endoscopic view of the defect edge. By repeating these steps, the apprentice endoscopist could become familiar with the correct distance between the clip and the defect edge, the appropriate direction of the clip and line, and skillful scope movement, thereby improving the learning curve.

**Fig. 2:**
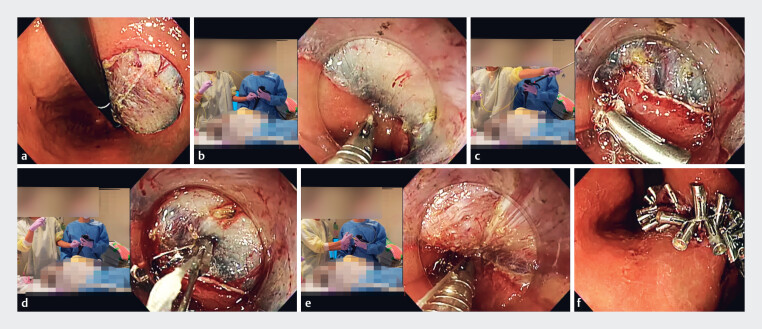
Reopenable clip-over-line method (ROLM) for endoscopic defect closure, using the master–apprentice technique to improve the learning curve. **a** A 40-mm mucosal defect after gastric endoscopic submucosal dissection (ESD). **b** The first clip with a line is placed in the distal mucosal defect. The master endoscopist manipulates the clip, and the apprentice endoscopist maneuvers the scope with guidance. **c** The master endoscopist indicates the position for the next clip placement. **d** Placement of the next reopenable clip at the contralateral edge of the defect; the line passing through the hole in one jaw of the clip can be seen. **e** With the defect edges grasped by the clips, the master endoscopist pulls the line by hand to bring the defect edges together firmly. As this procedure is repeated, apprentice endoscopists can learn the tips for the RCOL technique from the master endoscopist. **f** The mucosal defect from gastric ESD is completely closed.

Endoscopy_UCTN_Code_TTT_1AU_2AB

## Notes

Endoscopy E-Videos https://eref.thieme.de/e-videos
*E-Videos* is an open access online section of the journal *Endoscopy* , reporting on interesting cases and new techniques in gastroenterological endoscopy. All papers include a high-quality video and are published with a Creative Commons CC-BY license. Endoscopy E-Videos qualify for HINARI discounts and waivers and eligibility is automatically checked during the submission process. We grant 100% waivers to articles whose corresponding authors are based in Group A countries and 50% waivers to those who are based in Group B countries as classified by Research4Life (see: https://www.research4life.org/access/eligibility/ ). This section has its own submission website at https://mc.manuscriptcentral.com/e-videos .
